# The Preferential Therapeutic Potential of *Chlorella vulgaris* against Aflatoxin-Induced Hepatic Injury in Quail

**DOI:** 10.3390/toxins14120843

**Published:** 2022-12-01

**Authors:** Sawsan S. Elbasuni, Samar S. Ibrahim, Rasha Elsabagh, Mai O. Nada, Mona A. Elshemy, Ayman K. Ismail, Heba M. Mansour, Heba I. Ghamry, Samah F. Ibrahim, Ilhaam Alsaati, Ahmed Abdeen, Alshaimaa M. Said

**Affiliations:** 1 Department of Avian and Rabbit Diseases, Faculty of Veterinary Medicine, Benha University, Toukh 13736, Egypt; 2 Department of Forensic Medicine and Toxicology, Faculty of Veterinary Medicine, Benha University, Toukh 13736, Egypt; 3 Department of Food Hygiene and Control, Faculty of Veterinary Medicine, Benha University, Toukh 13736, Egypt; 4 Department of Veterinary Pharmacology, Animal Health Research Institute-Benha Branch, Benha 13518, Egypt; 5 Department of Clinical Pathology, Faculty of Veterinary Medicine, Benha University, Toukh 13736, Egypt; 6 Department of Forensic Medicine and Toxicology, Faculty of Veterinary Medicine, Suez Canal University, Ismailia 41522, Egypt; 7 Department of Pharmacology and Toxicology, College of Pharmaceutical Sciences and Drug Manufacturing, Misr University for Science and Technology, Giza 3236101, Egypt; 8 Department of Home Economics, College of Home Economics, King Khalid University, P.O. Box 960, Abha 61421, Saudi Arabia; 9 Department of Clinical Sciences, College of Medicine, Princess Nourah Bint Abdulrahman University, P.O. Box 84428, Riyadh 11671, Saudi Arabia; 10 Department of Basic Sciences, College of Medicine, Princess Nourah Bint Abdulrahman University, P.O. Box 84428, Riyadh 11671, Saudi Arabia; 11 Center of Excellence for Screening of Environmental Contaminants (CESEC), Benha University, Toukh 13736, Egypt; 12 Department of Biochemistry, Faculty of Veterinary Medicine, Benha University, Toukh 13736, Egypt

**Keywords:** *Chlorella vulgaris*, mycotoxins, oxidative stress, inflammatory cytokines, residues, meat quality

## Abstract

Aflatoxins (AFs) are the most detrimental mycotoxin, potentially hazardous to animals and humans. AFs in food threaten the health of consumers and cause liver cancer. Therefore, a safe, efficient, and friendly approach is attributed to the control of aflatoxicosis. Therefore, this study aimed to evaluate the impacts of *Chlorella vulgaris* (CLV) on hepatic aflatoxicosis, aflatoxin residues, and meat quality in quails. Quails were allocated into a control group; the CLV group received CLV (1 g/kg diet); the AF group received an AF-contaminated diet (50 ppb); and the AF+CLV group received both treatments. The results revealed that AF decreased the growth performance and caused a hepatic injury, exhibited as an increase in liver enzymes and disrupted lipid metabolism. In addition, AF induced oxidative stress, exhibited by a dramatic increase in the malondialdehyde (MDA) level and decreases in glutathione (GSH) level, superoxide dismutase (SOD), and glutathione peroxidase (GPx) activities. Significant up-regulation in the inflammatory cytokine (TNF-α, IL-1β, and IL-6) mRNA expression was also documented. Moreover, aflatoxin residues were detected in the liver and meat with an elevation of fat% alongside a decrease in meat protein%. On the other hand, CLV supplementation ameliorated AF-induced oxidative stress and inflammatory condition in addition to improving the nutritional value of meat and significantly reducing AF residues. CLV mitigated AF-induced hepatic damage, decreased growth performance, and lowered meat quality via its antioxidant and nutritional constituents.

## 1. Introduction

Aflatoxins (AFs) are the most predominant class of mycotoxins with the highest hazardous potential to humans and animals. AFs are released into the food and feed as a secondary metabolite of *Aspergillus flavus* and *A. parasiticus* [1,2]. In a favorable condition of this fungi growth, AFs contaminate the crop during production, harvesting, storage, and processing, resulting in building up hazardous concentrations. Worldwide climate change that occurs as a consequence of global warming provides optimal conditions for fungal growth as well as mycotoxin production [3]. It has been reported that AFs contaminate approximately 25% of the world’s crops [4]. In developing countries, around 4.5 billion people are in danger of chronic AF exposure [5]. The International Agency for Research on Cancer categorizes AF as a class I carcinogen. A growing body of literature has documented the hepatotoxic, immunosuppressive, and carcinogenic effects of AFs [2,6,7,8]. On the animal farm scale, AFs are known to significantly affect the performance of farm animals, including poultry, by lowering the growth rate, feed conversion, and meat production [9].

AFs constitute a serious health hazard due to their ability to accumulate in various tissues, particularly liver, where they are metabolized to a highly toxic reactive epoxide (mainly, aflatoxin-exo-8,9-epoxide; AFO), causing liver injury [10,11]. There is substantial evidence suggesting the occurrence of oxidative distress, DNA damage, inflammatory reactions, and apoptosis following AF exposure [2,7,8,12]. Moreover, to date, the stability of AFs and their metabolites against physical and chemical protocols used to reduce AF concentrations in food and feed may pose a health risk to consumers, elaborating the need for safe, feasible, and effective protocols for controlling AFs’ negative impacts [13]. The biological approaches using natural feed additives, such as curcumin [14], grape seed, or seabuckthorn [15], have been gaining attention recently. In addition, employing the beneficial micro-organisms in AF control is encouraging since they have antioxidant, anti-inflammatory, and immune-stimulating properties.

Recently, microalgae have been introduced in animal feed as a feasible replacement for fish meal, a good source of polyunsaturated fatty acids [16]. Moreover, it has been reported to have a valuable role in improving immunity, growth performance, and meat quality [17,18]. One of the green microalgae, *Chlorella vulgaris* (CLV), has an enormous nutritional value, since it is rich in various macro- and micronutrients. It contains polysaccharides, essential amino acids, essential fatty acids, more than 20 vitamins and minerals, and pigments [16]. Previous studies have attributed CLV’s antioxidant, hepato-protective, anti-inflammatory, and growth-promoting properties to its wide range of bioactive nutrients [17,19].

Quail production has recently witnessed a remarkable development, providing a new source of food security. Therefore, the current study was designed to investigate the potential use of CLV as a feed supplement of the biological source to minimize the hepatic toxic effects of AF on quails, reduce AF bioaccumulation, and improve meat quality. Consumers targeted quail meat for its low fat and cholesterol content, in addition to its high-biological-value protein, minerals, vitamins, and essential fatty acids [20].

## 2. Results

### 2.1. Growth Performance 

The impact of CLV supplementation on the growth performance of quails fed an AF-contaminated diet is presented in Table 1. The total body weight (BW) and body-weight gain (BWG) were significantly lowered in AF-exposed birds compared to all other treated ones and the highest BW and BWG were recorded in the CLV group. On the other hand, the AF+CLV group exhibited a marked increase in BWG compared to the AF group. Meanwhile, the feed conversion ratio (FCR) in the AF group was significantly higher than in other groups. On the other hand, the AF+CLV group exhibited a non-significant decrease in FCR compared to the AF group. Interestingly, the CLV group showed a significant decrease in FCR and a dramatic increase in productive performance parameters (BW, BWG, and survival rate) compared to the control group. In addition, no mortalities were observed in the control and CLV groups in contrast to AF-intoxicated quails and the AF+CLV group where the mortality rates were recorded at 26.67% and 3.33%, respectively. Consistently supplementing CLV to the AF diet promoted the AF-decreased survival rate (73.33%) up to 96.67%.

### 2.2. Changes in Liver Function Indices and Lipid Profile

**Figure 1:**
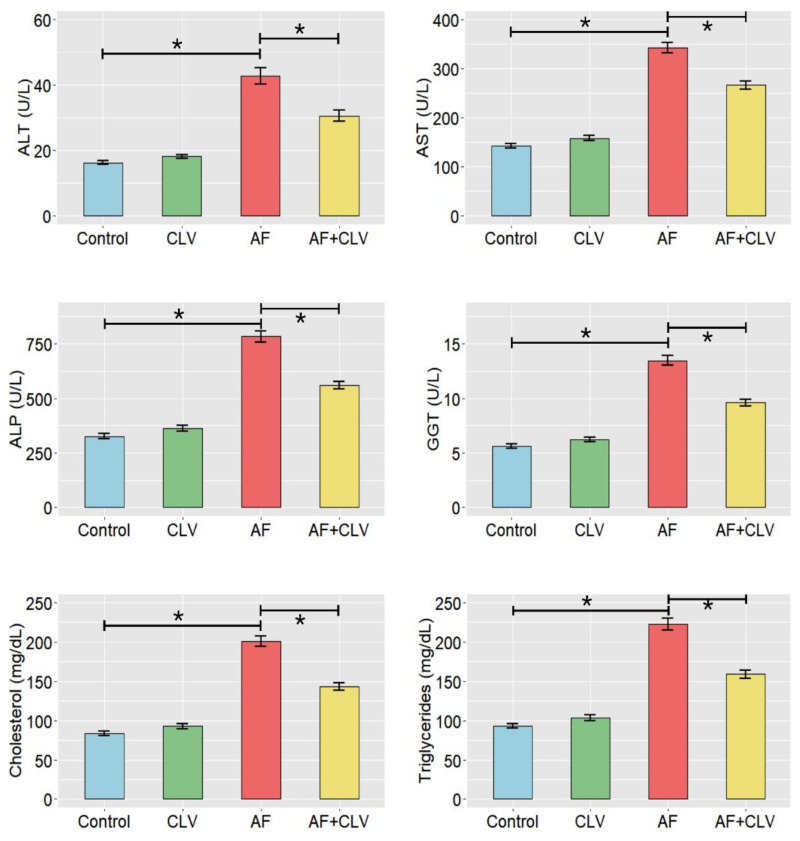
Bar plots of serum biochemical tests following *Chlorella vulgaris* and/or aflatoxin treatment. AF, aflatoxins; ALP, alkaline phosphatase; ALT, alanine aminotransferase; AST, aspartate aminotransferase; CLV, *Chlorella vulgaris*; GGT, gamma-glutamyl transpeptidase. Data are exhibited as mean ± SE (* *p* < 0.05).

According to the data presented in Figure 1, birds fed an AF-contaminated diet exhibited a noticeable increase in the activity of liver enzymes, including alanine aminotransferase (ALT), aspartate aminotransferase (AST), alkaline phosphatase (ALP), and gamma-glutamyl transpeptidase (GGT), when compared to those receiving basal diet (controls). On the other hand, adding CLV to an AF-contaminated diet showed amelioration in the activities of hepatic enzymes in the birds receiving that diet compared to those exposed to AF alone.

### 2.3. Changes in Hepatic Oxidant/Antioxidant Hemostasis

**Figure 2:**
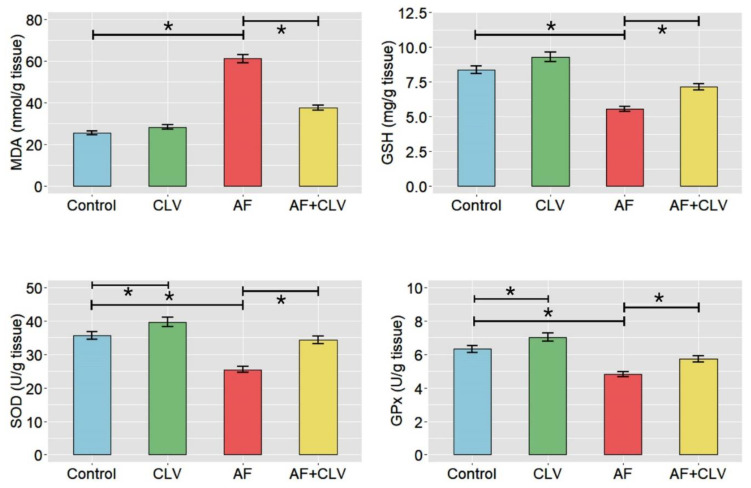
Bar plots of oxidant/antioxidant indices following *Chlorella vulgaris* and/or aflatoxin treatment in liver tissue. AF, aflatoxins; CLV, *Chlorella vulgaris*; GPx, glutathione peroxidase; GSH, reduced glutathione; MDA, malondialdehyde; SOD, superoxide dismutase. Data are exhibited as mean ± SE (* *p* < 0.05).

The study revealed that AF provoked a pronounced state of oxidative stress, as demonstrated by a dramatic increase in the hepatic malondialdehyde (MDA) and decrease in reduced-glutathione (GSH) levels, together with a noticeable reduction in the enzyme activities of the superoxide dismutase (SOD) and glutathione peroxidase (GPx) when compared to the control group. Of note, quails fed an AF plus CLV diet showed improvement in the alterations of MDA and GSH levels. The MDA level was decreased by 38.46% and GSH level was increased by 28.57% in the liver tissue of the AF+CLV group, as compared to the AF group. Moreover, the activities of hepatic antioxidant enzymes were almost brought back to normal levels. SOD activity was enhanced by 34.5% and GPx activity was increased by 19.23% in liver tissue, as matched to the AF sole treatment. Meanwhile, quails fed a CLV-supplemented diet exhibited a noticeable increase in antioxidant enzyme activities and GSH levels in the liver, as compared to the control group (Figure 2).

### 2.4. Changes in Inflammatory Cytokine mRNA Expressions

**Figure 3:**
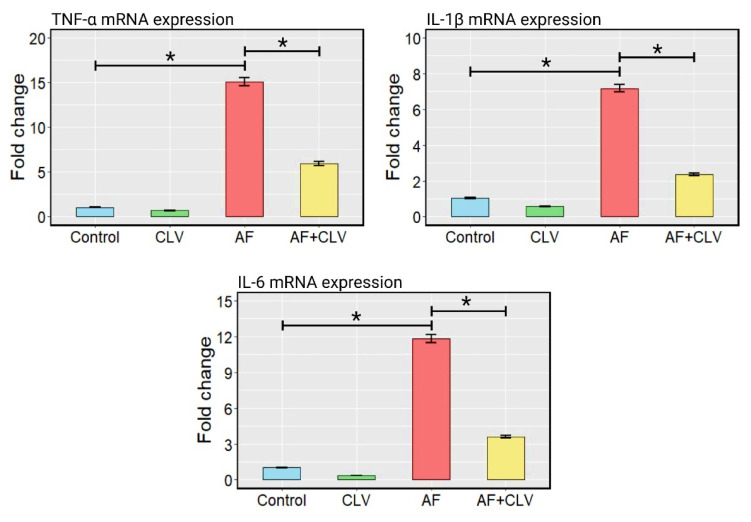
Bar plots of inflammatory cytokine mRNA expressions following *Chlorella vulgaris* and/or aflatoxin treatment in liver tissue. AF, aflatoxins; CLV, *Chlorella vulgaris*. Data are exhibited as mean ± SE (* *p* < 0.05).

As depicted in Figure 3, the AF could evoke an inflammatory reaction seen by drastic up-regulation of the inflammatory cytokine (TNF-α, IL-1β, and IL-6) mRNA expression levels in liver tissue compared to controls. However, adding CLV to the AF-contaminated diet could attenuate the AF-stimulated inflammatory response via down-regulation of the targeted inflammatory gene expressions.

### 2.5. Changes in Meat Nutritive Value after C. vulgaris and/or Aflatoxin Exposure

Results presented in Table 2 indicated that the CLV group exerted a significant increase in protein and a non-significant decrease in fat, cholesterol, and triacylglycerol contents of meat compared to controls. In comparison to the AF group, CLV supplementation to Japanese quails fed an AF-contaminated diet showed a marked increase in meat protein and a reduction in fat and cholesterol contents. Moreover, the triacylglycerols of meat were mostly brought back to the control level.

### 2.6. Impact of C. vulgaris on Aflatoxin Residues in Liver Tissue and Meat

Data shown in Table 2 revealed that quail fed an AF-contaminated diet exhibited a remarkable increase in AF residues in liver tissue and meat in comparison to birds receiving a basal diet. On the other hand, CLV supplementation could reduce the AF bioaccumulation in the liver and meat obtained from birds fed an AF-contaminated diet. However, no AF residues were detected in both groups fed on a basal diet or supplemented by CLV alone. The chromatograms of AF liberated from the HPLC are illustrated in Figures S1 and S2.

### 2.7. Hierarchical Clustering Heatmap and Variable Importance in Projection (VIP) Score 

**Figure 4:**
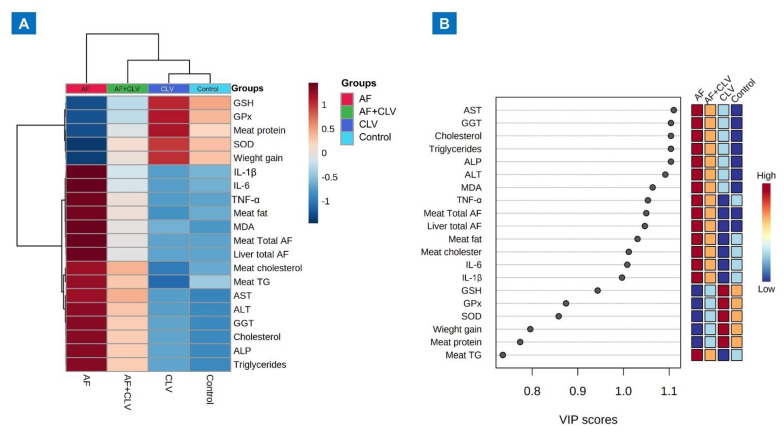
Multivariate analyses of all data sets following *Chlorella vulgaris* and/or aflatoxin treatment. (**A**) Hierarchical clustering heatmap summarizes all the data via an intuitive visualization. The concentration values are represented by each colored cell on the map, with varied averages in the rows and various treatment sets in the columns. On the gradation scale, dark red is the highest value and blue is the lowest. (**B**) Variable importance in projection (VIP) score, the relative concentrations of the pertinent measured parameters are shown in colored boxes on the right for each study group, and a colored scale from highest (red) to lowest (blue) indicates the contribution intensity (blue).

Then, multivariate analyses were performed to unravel the relationships between different parameters and treatments, as depicted in Figure 4. The clustering heatmap provides a clear visual of all the data sets in Figure 4A, highlighting the significant difference in concentration levels of all variables in response to AF toxicity in relation to the other treatments. Furthermore, the variable importance in projection (VIP) score indicated that AST, GGT, cholesterol, triglycerides, ALP, ALT, MDA, TNF-α, AF residue, IL-6, IL-1β, and GSH were the top influencing variables in our study, which were sensitive to different treatments and can discriminate AF treatment from others (Figure 4B).

### 2.8. Clinical, Postmortem, and Histopathological Examination

**Figure 5:**
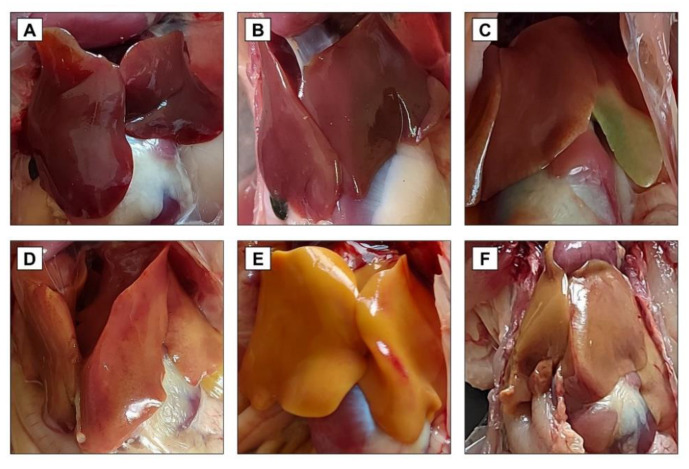
Macroscopic examination of liver following *Chlorella vulgaris* and/or aflatoxin treatment. (**A**) Control group; (**B**) CLV group; (**C**–**E**) AF group; and (**F**) AF+CLV group. AF, aflatoxins; CLV, *Chlorella vulgaris*.

**Figure 6:**
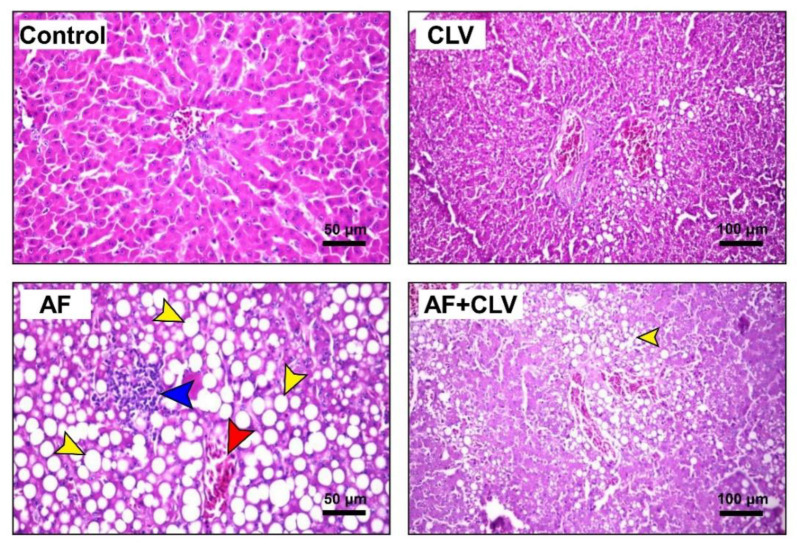
Microscopic examination of liver following *Chlorella vulgaris* and/or aflatoxin treatment. Sections from control (bar = 50 µm) and CLV (bar = 100 µm) groups displayed normal hepatocytes and central vein. Sections from AF group showed massive diffuse fatty degeneration with circumscribed vacuolated hepatocytes (yellow arrow), together with congestion in the central vein (red arrow), and mononuclear cell infiltration (blue arrow) (bar = 50 µm). Liver of AF+CLV group showed focal fatty degeneration (bar = 100 µm). AF, aflatoxins; CLV, *Chlorella vulgaris* (H & E stain).

During daily observations, the birds fed an AF-contaminated diet showed watery dropping, abnormal gait, and ruffled feathers. Macroscopically, the liver of AF-intoxicated birds appeared enlarged, friable, and light-yellow greenish in color, along with distended gall bladder and hemorrhages on the surface (Figure 5C–E). The histopathological examination indicated that the AF induced diffuse fatty degeneration in liver tissue with circumscribed vacuolated hepatocytes, congestion in the central vein, and focal mononuclear cell infiltration microscopically (Figure 6). On the other hand, CLV supplementation with the AF-contaminated diet significantly alleviated abnormal changes in liver tissue. Microscopically, CLV restored the damaged histological structure, except for focal degeneration in liver tissue (Figure 6). At most, birds in the AF+CLV group appeared healthy and alert with a normal gait, feather, and dropping.

## 3. Discussion

Our previous studies, along with others, strongly suggested the implication of oxidative stress and inflammation in AF-induced liver injury [2,7,8,12,21]. It is well known that the biotransformation of AF is catalyzed by hepatic cytochrome P450 elaborating more toxic metabolites, mainly aflatoxin-exo-8,9-epoxide (AFO). AFO has a strong affinity for electrons and can damage the liver by establishing irreversible covalent bonds with the nitrogen, oxygen, and sulphur heteroatoms found in biological macromolecules [22,23,24]. As a result, considerable amounts of free radicals are generated, such as O_2_^•−^, OH^•^, H_2_O_2_, and NO. These reactive radicals triggered lipid peroxidation (altered hepatocyte membrane integrity), mitochondrial dysfunction, protein misfolding, endoplasmic reticulum stress, exhaustion of cellular antioxidant competence, and formation of DNA adducts [2,25,26,27,28]. OH^•^ is the most injurious radical among others that can remotely attack the lipid bilayer in the cell membrane, causing lipid peroxidation, where the hepatocyte membrane loses its integrity, leading to the release of transaminases (ALT and AST), ALP, and GGT into the bloodstream, as indicated in the current study [23]. The documented increase in the level of MDA affirms the occurrence of lipid peroxidation in response to AF intoxication. MDA can also attack the distant cellular macromolecule, causing protein and DNA damage, making the matter worse [2]. The altered lipid profile could be attributed to the disruption of the biliary epithelium, as evidenced by increased serum GGT activity [29]. Furthermore, the AF–GSH complex is formed to eliminate the AF via urine. Prolonged AF exposure causes exhaustion of GSH capacity, in addition to the depletion of GPx, which is required for regeneration of GSH that leads to a depletion of the GSH store. The SOD activity was also reduced due to the overproduction of O_2_^•–^, since it is necessary for the dismutation of O_2_^•−^. Eventually, these events cause general perturbation of the cellular redox hemostasis, as indicated in the present study, wherein our current study along with previous findings strongly support the concept that oxidative stress and lipid peroxidation are the main modulatory mechanisms located behind AF-induced liver damage [2,7,8,12]. In addition, Zhang et al. recently reported the involvement of BACH1 in AF-induced oxidative stress and lipid peroxidation [30]. Sakamoto and his group also documented alterations in the quail performance and biochemical indices after exposure to AF [29]. The present gross and microscopical pathological findings confirmed the existence of severe damage after AF-exposed birds.

The current investigation offers compelling evidence for the hepato-protective potency of CLV against aflatoxicosis. Relevant studies have confirmed the protective efficacy of CLV against diazinon [19], deltamethrin [31], and sodium nitrite [32] toxicity. They attributed the ameliorative effect of CLV against hepatic toxicity of various toxicants to its antioxidant and ROS scavenging activities due to the vitamins and polyphenolic content [16]. Consistently, the observed improvements in liver function and oxidant/antioxidant state in the present investigation in birds co-administrated with AF and CLV might be due to the aforementioned reasons.

Moreover, the inflammatory condition observed in this study in response to AF intoxication is consistent with the previous results, which recorded up-regulation of inflammatory cytokine (TNF-α, IL-1β, and IL-6) gene expression in liver tissue in mice [2] or broiler chickens [33] exposed to AF. They attributed this up-regulation to the acute-phase response to inflammation. The modulatory role of oxidative stress in the inflammatory pathways cannot be ruled out. Excess ROS production stimulates the NF-κβ pathway, hence, inducing up-regulation of inflammatory cytokines, such as TNF-α, IL-1β, and IL-6, which was confirmed by the presence of lymphocytic infiltrations appearing after H&E staining. This was supported by the findings obtained by our group [2] and by Li et al., 2014 [34], where up-regulation of NF-κβ mRNA expression along with its downstream cytokines was reported. The current study supports CLV as an anti-inflammatory agent, as seen in the down-regulation of inflammatory cytokines. This is in good agreement with Abdelhamid et al., 2020 [19], who recorded the down-regulation of splenic TNF-α in a diazinon-intoxicated fish when supplemented with CLV. Suppressing the AF-induced inflammation might be attributed to the ROS scavenging potency of CLV and, consequently, inhibiting the NF-κβ signaling pathway [2,35].

A growing body of literature has evaluated the economic impact of different levels of aflatoxins regarding growth performance. Reductions in weight gain with deteriorations in FCR and productive efficiency were recorded after dietary AF inclusion in Japanese quails [29] as well as in broiler chickens [36], as observed in the present study. The adverse effect of AF on growth performance might be attributed to the negative nitrogen balance and impaired protein synthesis, gut health, and metabolic processes. Nitrogen intake is decreased, as the early sign of AF exposure is anorexia [36]. In addition, the oxidative deleterious impact and depletion of the cellular antioxidant competence of AF could be other possible mechanisms affecting the growth performance. It is worth mentioning that the results of our study emphasize the economic benefits of CLV as a growth promotor and production enhancer (Table 2). Such results are similar to those obtained by previous reports, which tested the beneficial impact of CLV on growth performance in quail [37], laying hens [38], and in rabbits [39]. A reasonable explanation is the digestibility and high-biological-value protein of CLV, which guarantees an increase in nitrogen intake, despite the feed intake [17]. Moreover, the polysaccharide content of CLV was noted to improve gut health via increasing lactic-acid-producing bacteria, which provides a suitable medium for optimal digestive performance [40].

The current data revealed that AF affected the nutritional value of meat obtained from the AF-exposed quails, unlike other treated groups. That was exhibited by deceased protein content and increased total fat, cholesterol, and triacylglycerols content. Since AF strongly causes DNA adducts and protein oxidation via the generated ROS, protein synthesis and lipid metabolism are disrupted [2,7,8]. The lipid composition of meat reflected the disturbing serum lipid profile in response to AF insult. Thus, it is hypothesized that these mechanisms might have a role in AF-altered nutritional value. As expected, high residuals of AF in liver and meat tissue were also documented in the current trial, which, in turn, affect the safety and quality of edible parts obtained from quail fed an AF-contaminated ration. Alternatively, supplementation of CLV enhanced the nutritive value of meat and reduced the AF residue in comparison to the AF group. Such improvements might relate to the antioxidant activity of CLV that could attenuate the toxic damage of AF, improving the liver function and, consequently, the metabolic processes. Moreover, CLV is an enriched source of high-quality nutrients, including essential amino acids, vitamins, and minerals correlated to its activity as a growth promotor and productive enhancer [41]. The effect of CLV on intestinal microflora played a crucial role in improving digestive efficiency, which would be another cause in improving the growth performance and nutritional value of quail meat [40]. These findings were in the same data frame as the previous reports [42,43,44].

**Figure 7:**
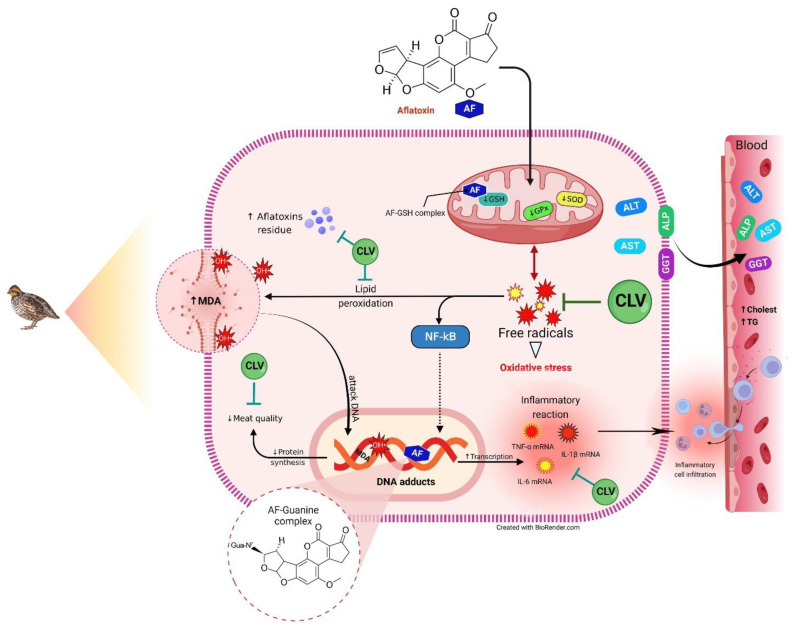
Molecular mechanisms behind the protective effect of CLV against AF-induced liver injury. AF, aflatoxins; ALP, alkaline phosphatase; ALT, alanine aminotransferase; AST, aspartate aminotransferase; CLV, *Chlorella vulgaris;* GGT, gamma-glutamyl transpeptidase GPx, glutathione peroxidase; GSH, reduced-glutathione; OH^•^, hydroxyl radical; IL-1β, interleukin-1β; IL-6, interleukin-6; MDA, malondialdehyde; NF-κB, nuclear factor kappa-B transcription factor; ROS, reactive oxygen species; SOD, superoxide dismutase; TNF-α, tumor necrosis factor-α.

Furthermore, multivariate statistical analyses, represented by clustering heatmap and VIP score, were performed to compile the variable contributions influenced by various treatments on liver tissue. The clustering heatmap clearly summarizes that AF exposure caused substantial changes in all studied parameters compared to other treated groups, suggesting potential improvements in those parameters when CLV was added. Additionally, the VIP score revealed that the top influencing variables in our study were AST, GGT, cholesterol, triglycerides, ALP, ALT, MDA, TNF-α, AF residue, IL-6, IL-1β, and GSH. Figure 7 underpins the molecular mechanisms behind the protective effect of CLV against AF-induced liver injury.

## 4. Conclusions

AF evoked a remarkable hepatic dysfunction and disrupted the metabolism via the induction of oxidative damage, lipid peroxidation, and inflammatory reactions, lowering the meat nutritional value and increasing the AF tissue residues. CLV supplementation has the ability to protect the hepatocytes from the injurious impact of AF, which might be CLV’s antioxidant, ROS-scavenging, and anti-inflammatory activities, along with its enriched nutritive value. We anticipate that CLV supplementation could be an efficient, safe, and feasible biological procedure, counteracting the hazardous impact of AF on animals and humans.

## 5. Materials and Methods

### 5.1. Experimental Design

Ten-week-old Japanese quails were purchased from the Faculty of Veterinary Medicine, Benha University, Egypt. Quails were subjected to 17 h of light per day during the study and were fed a commercial corn and soybean meal basal diet that meets all the nutritional requirements for quails according to specifications of the NRC (1994) and water was provided ad libitum.

**Figure 8:**
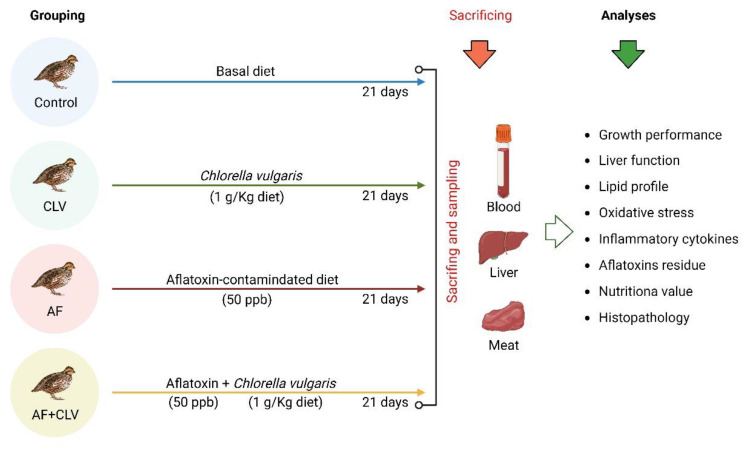
Experimental design.

After an acclimatization period of two weeks, the birds were allocated into four groups with three replicates of five birds each. The experimental groups include: control group, received a basal diet, *C. vulgaris* group (CLV), received a basal diet supplemented with CLV (1 g/kg) [17], aflatoxin group (AF), received an AF-contaminated diet (50 ppb; Aflatoxin mix, purity > 98%, Merk, Darmstadt, Germany), and aflatoxin and *C. vulgaris* group (AF+CLV), received AF-contaminated diet supplemented with CLV for three weeks (Figure 8). Birds in all experimental groups were reared under the same management, hygienic, and environmental conditions.

### 5.2. Growth Performance Assessment

The weight of birds was recorded at the beginning and end of the experiment to calculate body-weight gain (BWG). The feed conversion ratio (FCR) was calculated from feed intake (kg) in relation to the BWG (kg). Clinical symptoms were observed in all experimental groups and the mortality and survival rates were recorded.

### 5.3. Biochemical Analyses

At the end of the experiment, blood samples were collected from the jugular vein and sera were harvested and stored at −20 °C for further biochemical analysis. Liver enzymes included ALT, AST, ALP, and GGT activities. Serum total cholesterol and triacylglycerols were also determined. All procedures were carried out according to the manufacturers’ instructions (Laboratory Biodiagnostics, Cairo, Egypt).

Liver tissue was collected from the humanely slaughtered bird and stored at −80 °C until used for determination of tissue oxidation indices spectrophotometrically and inflammatory markers via the qRT-PCR technique. Moreover, the MDA and GSH levels, in addition to the SOD and GPx activities, were evaluated following the manufacturer manual (Laboratory Biodiagnostics).

### 5.4. Quantitative Real-Time PCR (qRT-PCR)

The total RNA was extracted using RNeasy Mini Kit (Cat#74104, QIAGEN Sciences Inc., Germantown, MD, USA) following the manufacturer’s procedures. The used primer sequences of the targeted genes (inflammatory cytokines (TNF-α, IL-1β, and IL-6) and 28S rRNA, a housekeeping gene) are listed in Table 3. The qRT-PCR was executed using QuantiTect probe RT-PCR (Cat#204443, QIAGEN Sciences Inc.). The cycling condition of PCR was conducted at 50 °C for 30 min, 94 °C for 10 min, 40 cycles at 94 °C for 15 s, and 60 °C for 1 min using a real-time PCR machine (Applied Biosystems, Waltham, CA, USA). The Stratagene MX3005P software determined the amplification curves and cycle threshold (Ct) values. Fold changes in the expression levels were calculated using the 2^−ΔΔCt^ method and after normalization against the housekeeping gene.

### 5.5. Evaluation of Meat Nutritive Value

Total protein and fat content in meat were evaluated following the method of Anderson, 2007 [48]. The spectrophotometric estimation of cholesterol and triacylglycerols contents was also performed as described by El-Medany and El-Reffaei, 2015 [49].

### 5.6. Aflatoxin Residues in Liver Tissue and Meat

Liver tissues and meat were collected from the slaughtered birds at the end of the trial to determine the AF residue using high-performance liquid chromatography. The extraction and analysis were carried out as described by Abdel-Monem et al., 2015 [50]. Aflatoxin B_1_, B_2_, G_1_, and G_2_ reference materials (Supelco^®^, Merk) were employed in those analyses.

### 5.7. Postmortem and Histopathological Examination

The sacrificed birds were immediately examined for postmortem lesions with a special focus on liver. Parts from the liver were gathered and immediately fixed in 10% formalin for at least 24 h. The fixed specimens were processed for histopathological examination following the standard protocols. Sections of 4 µm thickness were cut and stained with hematoxylin and eosin (H & E), examined under a light microscope, and imaged using a digital-camera-integrated system.

### 5.8. Statistical Analyses

The obtained data were analyzed using a one-way analysis of variance (ANOVA) followed by LSD as the post hoc test. The analysis was performed using SPSS 25 software for Windows (SPSS Inc., Chicago, IL, USA). Data were expressed as the mean ± SE. The data were statistically significant at *p* values < 0.05. Moreover, a clustering heatmap and variable importance projection (VIP) score were generated by RStudio under R version 4.0.2.

## Figures and Tables

**Table 1:** Impact of *Chlorella vulgaris* supplementation on aflatoxin-altered growth performance.

Parameters	Experimental Groups			
	Control	CLV	AF	AF+CLV
BW (g)	265.7 ± 27.5	286.7 ± 34.3	218.0 ± 16.5 *	257.7 ± 25.5 ^#^
BWG (g)	15.67 ± 2.3	36.67 ± 6.8	(-)32 ± 16 *	7.67 ± 4.2 ^#^
FCR	3.82 ± 0.04	3.18 ± 0.19 *	5.15 ± 0.13 *	4.54 ± 0.31 *
Mortality %	0	0	26.67%	3.33%
Survival rate (%)	100%	100%	73.33%	96.67%

AF; aflatoxins, BW; body weight, BWG; body-weight gain, CLV; *Chlorella vulgaris*, FCR; feed conversion ratio. Data are presented as mean ± S.E. * *p* < 0.05 vs. Control group, # *p* < 0.05 vs. AF group.

**Table 2:** Impact of *Chlorella vulgaris* supplementation on meat nutritive value and total aflatoxin residue.

Parameters	Experimental Groups			
	Control	CLV	AF	AF+CLV
Nutritive value				
Protein%	22.76 ± 0.56	24.94 ± 0.67 *	19.54 ± 0.47 *	22.19 ± 0.48 ^#^
Fat%	0.57 ± 0.01	0.53 ± 0.01	1.07 ± 0.03 *	0.74 ± 0.02 *^#^
Cholesterol (mg/g)	20.66 ± 0.51	19.44 ± 0.52	27.74 ± 0.66 *	24.65 ± 0.53 *^#^
Triacylglycerols (mg/100 g)	58.75 ± 1.45	56.79 ± 1.52	63.56 ± 1.52 *	60.77 ± 1.31
AF residue (ppb)				
Liver	ND	ND	121.54± 2.60 *	43.32 ± 0.81 *^#^
Meat	ND	ND	76.98± 1.65 *	28.21 ± 0.53 *^#^

AF; aflatoxin, CLV; *Chlorella vulgaris*, ND; not detected. Data are presented as mean ± S.E. * *p* < 0.05 vs. Control group, # *p* < 0.05 vs. AF group.

**Table 3:** Primers used for qRT-PCR.

Gene	Primer Sequence	Reference
28S rRNA	F: GGCGAAGCCAGAGGAAACT R: GACGACCGATTTGCACGTC	[45]
TNF-α	F: CCCCTACCCTGTCCCACAA R: ACTGCGGAGGGTTCATTCC	[46]
IL-1β	F: GCTCTACATGTCGTGTGTGATGAG R: TGTCGATGTCCCGCATGA	[47]
IL-6	F: GCTCGCCGGCTTCGA R: GGTAGGTCTGAAAGGCGAACAG	[45]

## Data Availability

Upon request, the data utilized to verify the findings of this research are obtainable from the corresponding authors.

## Supplementary Materials

The following supporting information can be downloaded at: https://www.mdpi.com/article/10.3390/toxins14120843/s1, Figure S1: A representative chromatogram of AF detected in liver tissue; Figure S2: A representative chromatogram of AF detected in meat.
