# Supplementary material for: The Preferential Therapeutic Potential of Chlorella vulgaris against Aflatoxin-Induced Hepatic Injury in Quail
Source: Toxins (Basel). 2022 Dec 1;14(12):843. doi: 10.3390/toxins14120843 (PMC9787596; doi:10.3390/toxins14120843)
Supplement: Supplementary file 1 [file toxins-14-00843-s001.zip › toxins-2053811-supplementary.pdf]

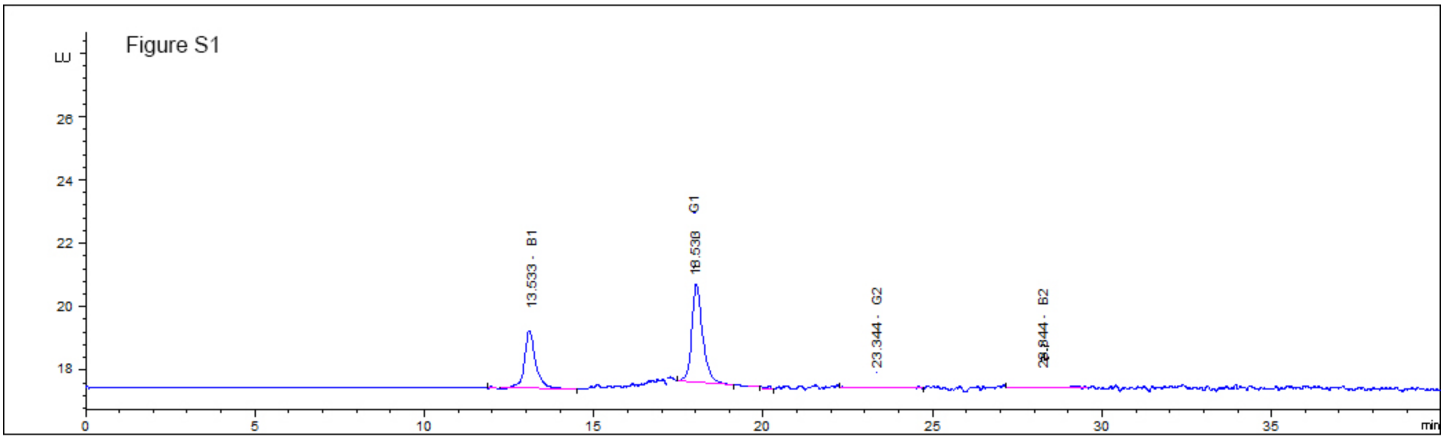

Figure S1: A representative chromatogram of AF detected in liver tissue

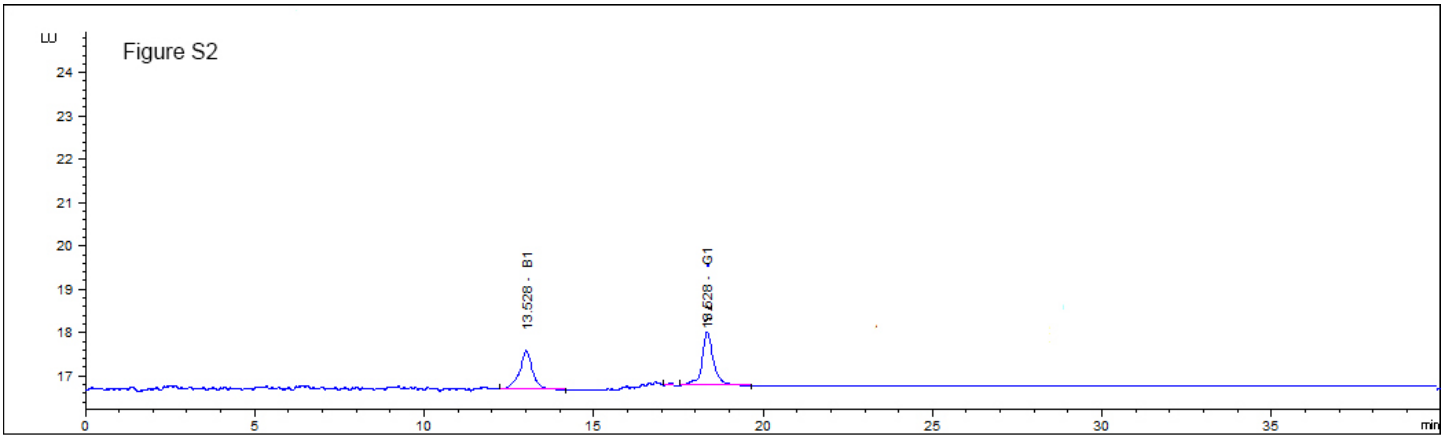

Figure S2: A representative chromatogram of AF detected in meat
